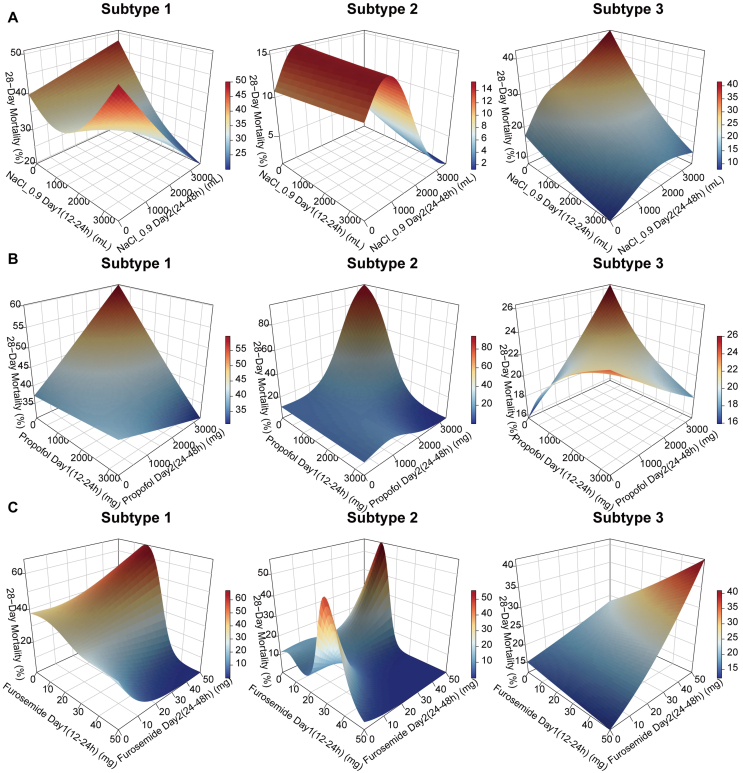# Identification of vital sign trajectory phenotypes and treatment response heterogeneity in critically ill patients with ischemic stroke: A multicenter study with external validation

**DOI:** 10.1016/j.neurot.2026.e00896

**Published:** 2026-04-08

**Authors:** Lijuan Wang, Duozi Wang, Bo Tang, Jun Duan, Jie Huang

**Affiliations:** a Department of Neurology, Sichuan Provincial People's Hospital, University of Electronic Science and Technology of China, Chengdu, Sichuan 610072, China; b School of Medicine, University of Electronic Science and Technology of China, Chengdu, Sichuan 610072, China

**Keywords:** Ischemic stroke, Critical care, Vital sign trajectory, Phenotyping, Treatment heterogeneity

## Abstract

Critically ill patients with ischemic stroke exhibit heterogeneous hemodynamic patterns, yet previous trajectory-based studies have focused on single vital sign parameters. We conducted a multicenter retrospective cohort study to identify vital sign trajectory phenotypes, evaluate their prognostic value compared with traditional severity scores, and explore treatment response heterogeneity. Using group-based multi-trajectory modeling of six vital signs (systolic blood pressure, diastolic blood pressure, mean arterial pressure, heart rate, respiratory rate, and oxygen saturation) during the first 12 h after intensive care unit admission, we analyzed 3100 patients from MIMIC-IV for development and 3951 patients from eICU and Chinese Critical Care Database for external validation. Three distinct phenotypes were identified: tachycardic-tachypneic (25.6%), hypertensive-stable (37.5%), and low-diastolic quiescent (36.8%), with in-hospital mortality rates of 29.7%, 5.9%, and 14.9%, respectively. After multivariable adjustment, the tachycardic-tachypneic phenotype demonstrated significantly elevated in-hospital mortality risk compared with hypertensive-stable (HR 3.55, 95% CI 2.23–5.64), with consistent associations for 28-day (HR 4.38, 95% CI 2.97–6.46) and one-year mortality (HR 4.50, 95% CI 3.35–6.04). Phenotype-based classification outperformed SOFA, SAPS II, and Charlson index for one-year mortality prediction. Exploratory analysis revealed phenotype-specific dose-mortality associations for normal saline volumes, with tachycardic-tachypneic and hypertensive-stable phenotypes showing lower predicted mortality at higher Day 1 vol (2357–3500 mL and 1929–3500 mL), while low-diastolic quiescent showed lower predicted mortality with restricted Day 2 fluids (0–714 mL). Propofol similarly showed differential dose-mortality associations across phenotypes. These findings support trajectory-based phenotyping for early risk stratification and may inform precision-guided therapeutic approaches in critically ill ischemic stroke patients.

## Graphical abstract

**Figure:**
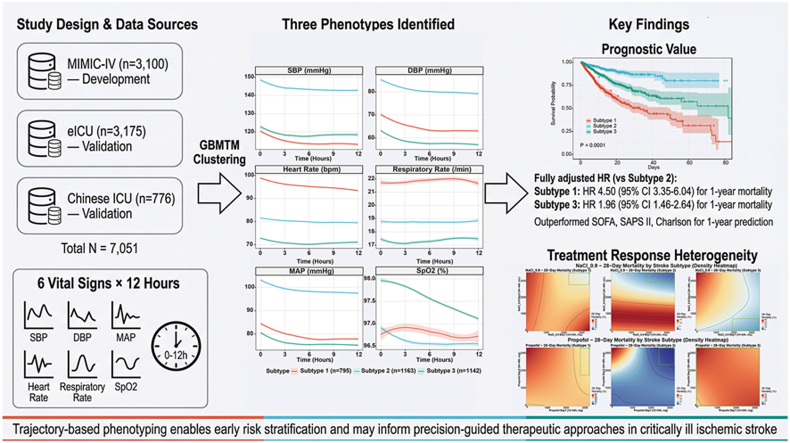

## Introduction

Ischemic stroke remains one of the leading causes of disability and mortality worldwide, accounting for approximately two-thirds of all stroke cases and imposing substantial burdens on patients, families, and healthcare systems [1]. Among patients admitted to intensive care units (ICUs), those with ischemic stroke represent a particularly vulnerable population characterized by hemodynamic instability, heightened inflammatory responses, and elevated risks of adverse outcomes including in-hospital mortality, prolonged mechanical ventilation, and multiorgan dysfunction [2]. The heterogeneous clinical presentations and unpredictable trajectories of critically ill stroke patients pose significant challenges for risk stratification and individualized treatment decision-making [3].

Over the past decade, accumulating evidence has demonstrated the prognostic significance of vital sign dynamics in acute ischemic stroke. Studies have established that blood pressure trajectories in the first 24 h after endovascular thrombectomy are associated with neurological deterioration, intracranial hemorrhage, and functional outcomes [4]. Similarly, one-year systolic blood pressure trajectories have been linked to composite cardiovascular outcomes including stroke recurrence, myocardial infarction, and all-cause mortality [5]. Beyond blood pressure, heart rate trajectories during the acute phase have been identified as predictors of 3-month functional outcomes in ischemic stroke patients [6]. Furthermore, group-based trajectory modeling has been successfully applied to characterize longitudinal patterns of fluid balance [7], blood glucose [8], serum lactate [9], and serum sodium [10] in critically ill stroke populations, revealing distinct subgroups with differential mortality risks.

However, previous studies have predominantly focused on single vital sign parameters or specific biomarkers, neglecting the multidimensional nature of hemodynamic monitoring in ICU settings. The interplay among multiple vital signs—including blood pressure, heart rate, respiratory rate, and oxygen saturation—may better capture the complex pathophysiological states underlying clinical deterioration. Additionally, most trajectory-based studies have been limited to single-center cohorts without external validation, raising concerns about generalizability across diverse populations and healthcare systems. Whether distinct multivariable vital sign trajectory phenotypes exist in critically ill ischemic stroke patients, and whether these phenotypes demonstrate heterogeneous responses to common ICU treatments, remains largely unexplored.

Therefore, we conducted a multicenter retrospective cohort study utilizing three large critical care databases—MIMIC-IV, eICU, and a Chinese critical care database—to develop and externally validate vital sign trajectory-based phenotypes in critically ill patients with ischemic stroke. Using group-based multi-trajectory modeling, we aimed to identify distinct phenotypes based on six vital sign parameters during the first 12 h after ICU admission, evaluate their prognostic value for short-term and long-term mortality, and explore treatment response heterogeneity across phenotypes to inform precision critical care management.

## Materials and Methods

### Study design and data sources

This multicenter retrospective observational cohort study utilized data from three critical care databases to develop and externally validate a vital sign trajectory-based phenotyping model for critically ill patients with ischemic stroke. The primary analysis was conducted using the Medical Information Mart for Intensive Care IV (MIMIC-IV, version 2.2), a publicly available database containing deidentified health records from patients admitted to intensive care units at Beth Israel Deaconess Medical Center between 2008 and 2019 [11]. External validation was performed using the eICU Collaborative Research Database (version 2.0), which comprises data from over 200 hospitals across the United States collected through the Philips eICU telehealth program between 2014 and 2015 [12], and a Chinese critical care database established from electronic healthcare records at a tertiary care medical center in China from January 2012 to May 2022 [13]. This study was conducted in accordance with the principles of the Declaration of Helsinki and followed the Strengthening the Reporting of Observational Studies in Epidemiology (STROBE) guidelines and the Transparent Reporting of a Multivariable Prediction Model for Individual Prognosis or Diagnosis (TRIPOD) guidelines.

### Study population

Adult patients aged 18 years or older with a diagnosis of ischemic stroke were included in this study. Ischemic stroke was identified using International Classification of Diseases codes (ICD-9: 433. x, 434. x, 436. x; ICD-10: I63. x). For patients with multiple ICU admissions, only the first ICU stay was included. Patients were excluded if they had an ICU length of stay less than 24 h, missing vital sign measurements during the first 12 h after ICU admission, or incomplete outcome data. After applying these criteria, the final cohort consisted of patients from MIMIC-IV for model development, with eICU and the Chinese critical care database serving as external validation cohorts.

### Vital sign trajectory variables and data preprocessing

Six vital sign parameters were selected as trajectory variables for phenotyping based on their clinical relevance to hemodynamic monitoring in ischemic stroke: systolic blood pressure (SBP), diastolic blood pressure (DBP), mean arterial pressure (MAP), heart rate, respiratory rate, and peripheral oxygen saturation (SpO2). Vital signs were extracted at hourly intervals from 0 to 12 h after ICU admission, resulting in 13 time points per patient.

### Group-based multi-trajectory modeling

Patient phenotypes were identified using group-based multi-trajectory modeling (GBMTM), a method that simultaneously models multiple longitudinal trajectories to identify distinct subgroups with similar temporal patterns across all variables. The GBMTM was implemented using the flexmix package in R, with polynomial terms (linear and quadratic) included to capture nonlinear temporal trends. Models with cluster numbers ranging from 2 to 7 were evaluated, and the optimal number of clusters was determined based on a comprehensive evaluation of multiple criteria, including the Akaike Information Criterion (AIC), Bayesian Information Criterion (BIC), average silhouette coefficient, Calinski-Harabasz index, and gap statistic, with consideration given to minimum cluster size (at least 5% of the total population) and model convergence. Clustering quality was assessed using silhouette coefficient, Calinski-Harabasz index, and Davies-Bouldin index. Bootstrap resampling with 1000 iterations was performed to evaluate cluster stability using Jaccard similarity coefficients. Principal component analysis was applied for dimensionality reduction and visualization of cluster separation.

### Outcome definitions

The primary outcome was in-hospital mortality, defined as death occurring during hospitalization. Secondary outcomes included 28-day mortality, one-year mortality, and functional status at discharge assessed by the modified Rankin Scale (MRS). Time-to-event outcomes were calculated from ICU admission to death or censoring at discharge or the end of the follow-up period.

### Statistical analysis

Baseline characteristics were compared across the three databases using standardized mean differences, with categorical variables presented as frequencies and percentages and continuous variables as medians with interquartile ranges. Stroke-specific characteristics including National Institutes of Health Stroke Scale (NIHSS) score, large vessel occlusion (LVO) status, endovascular thrombectomy (EVT), and Trial of ORG 10172 in Acute Stroke Treatment (TOAST) classification were extracted and compared across databases. Survival analysis was performed using Kaplan-Meier curves with log-rank tests for comparison between phenotypes. The distribution of MRS scores at discharge was compared across phenotypes using Kruskal-Wallis tests with Dunn's pairwise comparisons (Bonferroni-corrected). Cox proportional hazards regression was conducted to estimate hazard ratios with 95% confidence intervals for all mortality outcomes (in-hospital, 28-day, and one-year mortality), using the phenotype with the lowest mortality rate as the reference group. Four progressive multivariable models were constructed: crude model, model adjusted for demographics (age, sex, BMI), model further adjusted for severity scores (SOFA, SAPS II, Charlson comorbidity index, Glasgow Coma Scale), and fully adjusted model additionally including laboratory values (first 24-h creatinine, blood urea nitrogen, blood glucose, and potassium) and interventions (mechanical ventilation, vasopressor use, sedative use, and renal replacement therapy).

To generate predicted probabilities for discrimination analysis, phenotype membership was entered as a dummy variable into logistic regression models. Discriminative performance was evaluated using the area under the receiver operating characteristic curve (AUROC) and the area under the precision-recall curve (AUPRC) to account for class imbalance, with 95% confidence intervals. Comparisons between phenotype-based prediction and traditional severity scores (SOFA, SAPS II, OASIS, Charlson) were performed using DeLong's test. Incremental predictive value was assessed using net reclassification improvement (NRI) and integrated discrimination improvement (IDI) with bootstrap confidence intervals. Calibration was assessed by plotting predicted versus observed probabilities. Decision curve analysis was performed to evaluate the clinical utility of phenotype-based prediction by quantifying net benefit across a range of threshold probabilities.

To evaluate the robustness of the phenotype-mortality association, subgroup analyses were performed across 14 clinical subgroups stratified by demographics (sex, age, BMI), stroke characteristics (NIHSS, LVO, EVT, TOAST classification), disease severity (SOFA, GCS, Charlson comorbidity index), and interventions (mechanical ventilation, renal replacement therapy, vasopressor use, sedative use). Hazard ratios were estimated using Cox proportional hazards models within each subgroup, and interaction effects were assessed using likelihood ratio tests.

### Treatment response heterogeneity analysis

To explore associations between treatment doses and mortality across phenotypes, generalized additive models (GAM) with tensor product smooths were fitted for each phenotype separately, adjusting for demographics (age, sex, BMI), severity scores (SOFA, SAPS II, Charlson comorbidity index, Glasgow Coma Scale), laboratory values (first 24-h creatinine, blood urea nitrogen, blood glucose, and potassium), interventions (mechanical ventilation, vasopressor use, sedative use, and renal replacement therapy), and stroke-specific characteristics (NIHSS, LVO, EVT, TOAST classification)∗∗. Three treatments were analyzed: normal saline (0.9% NaCl), propofol, and furosemide. The models examined the association between cumulative doses administered during Day 1 (12–24 h after ICU admission) and Day 2 (24–48 h after ICU admission) with 28-day mortality. The treatment exposure windows were defined to start after the 12-h vital sign trajectory ascertainment period to ensure temporal separation between phenotype definition and treatment exposure. Predicted mortality surfaces were visualized using three-dimensional surface plots and two-dimensional density heatmaps, with the lowest 5% of predicted mortality regions identified as areas of interest.

### External validation

External validation was performed by applying the GBMTM phenotypes developed in MIMIC-IV to the eICU and Chinese databases. Standardization parameters (mean and standard deviation) derived from the MIMIC-IV training data were used to normalize the external datasets. Each patient in the validation cohorts was assigned to the phenotype with the minimum trajectory mean squared error relative to the phenotype centroids. Phenotype distributions, trajectory patterns, and survival outcomes were compared across databases to assess generalizability.

All statistical analyses were performed using R version 4.3.0 with packages including flexmix, survival, survminer, pROC, mgcv, and forestploter. A two-sided P value less than 0.05 was considered statistically significant.

## Results

### Study population and baseline characteristics

A total of 11,214 patients diagnosed with ischemic stroke were initially identified from the three databases, including 5,294 from MIMIC-IV, 4,862 from eICU, and 1,058 from the Chinese critical care database. After applying the exclusion criteria, 7,051 patients were included in the final analysis: 3100 from MIMIC-IV for model development, and 3,175 from eICU and 776 from the Chinese database for external validation (Fig. 1). The primary reasons for exclusion were non-first hospital admissions and multiple ICU admissions (n = 2816), ICU length of stay less than 24 h (n = 250), and missing vital signs data within the first 12 h (n = 1094).

**Fig. 1:**
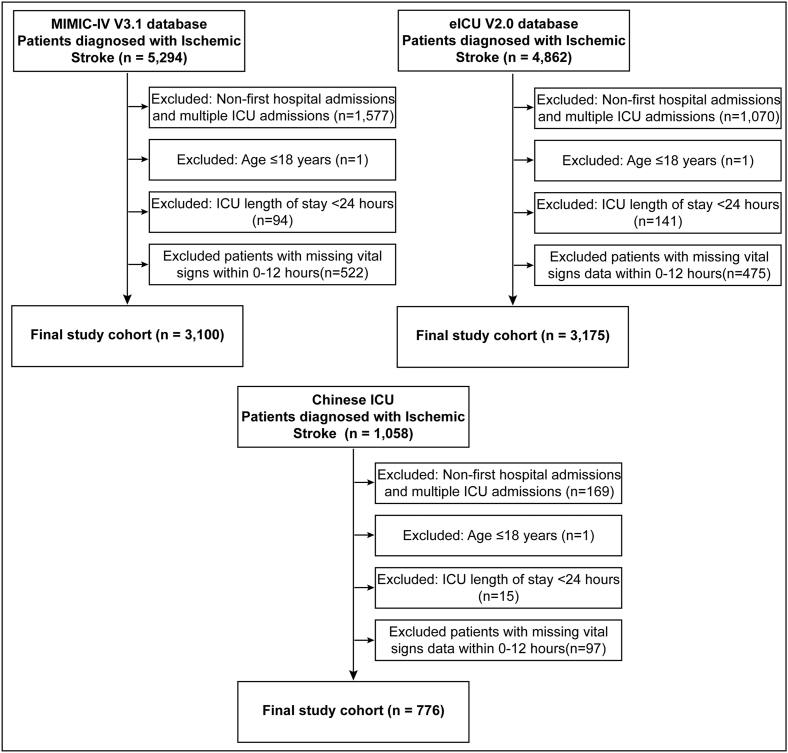
**Study Population Flowchart.** Flowchart illustrating the patient selection process from the three databases (MIMIC-IV, eICU, and Chinese critical care database), including inclusion and exclusion criteria and the final sample sizes for the development and validation cohorts.

The baseline characteristics of the study population are presented in Table 1. The median age ranged from 70.00 years in eICU to 73.86 years in the Chinese cohort, with male patients accounting for approximately half of the population across all databases (51.2%–53.5%). The median NIHSS score was highest in the Chinese cohort (15.00 [11.00, 18.00]) and lowest in eICU (12.00 [8.00, 15.00]; SMD = 0.368). Large vessel occlusion was identified in 41.6% of MIMIC-IV, 34.6% of eICU, and 50.1% of Chinese cohort patients (SMD = 0.212). Endovascular thrombectomy was performed in 11.0%, 7.5%, and 8.9% of patients, respectively (SMD = 0.080). TOAST classification revealed that large artery atherosclerosis was the predominant subtype in the Chinese cohort (39.4%), while cardioembolism and large artery atherosclerosis were similarly prevalent in MIMIC-IV (29.9% and 30.0%) and eICU (29.6% and 26.6%). Significant differences were observed in disease severity scores among the three cohorts. The Charlson comorbidity index showed the largest standardized mean difference (SMD = 0.743), with MIMIC-IV patients having the highest burden (7.00 [5.00, 9.00]). Regarding interventions, mechanical ventilation rates varied substantially from 27.0% in eICU to 46.6% in the Chinese cohort, while vasopressor use was most prevalent in the Chinese database (33.6%) and lowest in eICU (9.9%). The distribution of modified Rankin Scale scores at discharge differed significantly across databases (P < 0.001, SMD = 0.394), with the Chinese cohort exhibiting the highest proportion of severe disability (MRS 4–5: 52.1%) and mortality (MRS 6: 24.2%). The in-hospital mortality rates differed significantly, with the highest observed in the Chinese cohort (24.2%), followed by MIMIC-IV (15.3%) and eICU (11.6%). The temporal trends of the six trajectory variables during the first 12 h after ICU admission demonstrated consistent patterns across databases (Fig. 2, Supplementary Table 1). Systolic blood pressure showed a declining trend from baseline (MIMIC-IV: 132.43 mmHg; eICU: 139.57 mmHg; Chinese: 134.78 mmHg) and stabilized after approximately 6 h. Heart rate and respiratory rate remained relatively stable throughout the observation period, while SpO2 maintained above 96% across all cohorts.

**Table 1:** Baseline characteristics of ischemic stroke patients across three databases.

Characteristics	Level	MIMIC-IV (n = 3100)	eICU (n = 3175)	ChineseICU (n = 776)	P value	SMD
Age, years		72.00 [62.00, 81.00]	70.00 [59.00, 80.00]	73.86 [63.02, 81.94]	<0.001	0.115
Gender	Male	1657 (53.5)	1629 (51.3)	397 (51.2)	0.193	0.031
	Female	1443 (46.5)	1546 (48.7)	379 (48.8)		
Body mass index, kg/m^2^		27.30 [24.00, 31.48]	27.61 [24.01, 31.96]	27.41 [23.70, 31.63]	0.170	0.044
NIHSS score		14.00 [10.00, 18.00]	12.00 [8.00, 15.00]	15.00 [11.00, 18.00]	<0.001	0.368
Large vessel occlusion	No	1809 (58.4)	2078 (65.4)	387 (49.9)	<0.001	0.212
	Yes	1291 (41.6)	1097 (34.6)	389 (50.1)		
Endovascular thrombectomy	No	2759 (89.0)	2936 (92.5)	707 (91.1)	<0.001	0.080
	Yes	341 (11.0)	239 (7.5)	69 (8.9)		
TOAST classification	Large artery atherosclerosis	929 (30.0)	845 (26.6)	306 (39.4)	<0.001	0.223
	Cardioembolism	927 (29.9)	940 (29.6)	196 (25.3)		
	Small vessel occlusion	483 (15.6)	583 (18.4)	78 (10.1)		
	Other determined	112 (3.6)	120 (3.8)	33 (4.3)		
	Undetermined	649 (20.9)	687 (21.6)	163 (21.0)		
SAPS II score		34.00 [26.00, 43.00]	27.00 [21.00, 37.00]	34.00 [27.00, 43.00]	<0.001	0.304
OASIS score		31.00 [25.00, 37.00]	26.00 [21.00, 33.00]	33.00 [26.75, 39.00]	<0.001	0.431
SOFA score		3.00 [2.00, 6.00]	4.00 [3.00, 6.00]	3.00 [2.00, 5.00]	<0.001	0.236
Glasgow coma scale		14.00 [13.00, 15.00]	13.80 [10.00, 15.00]	15.00 [13.00, 15.00]	<0.001	0.253
Charlson comorbidity index		7.00 [5.00, 9.00]	4.00 [2.00, 5.00]	6.00 [5.00, 7.00]	<0.001	0.743
Heart rate, bpm		81.00 [70.00, 95.00]	80.00 [69.00, 93.00]	80.00 [67.00, 93.00]	<0.001	0.081
Systolic BP, mmHg		133.00 [116.00, 150.00]	142.00 [124.00, 160.00]	135.00 [116.00, 154.00]	<0.001	0.213
Diastolic BP, mmHg		73.00 [60.00, 87.00]	75.00 [63.00, 87.00]	63.00 [53.00, 75.00]	<0.001	0.417
Mean arterial pressure, mmHg		91.00 [78.00, 104.00]	94.00 [81.00, 108.00]	87.00 [75.00, 100.67]	<0.001	0.240
Temperature, °C		36.72 [36.50, 37.00]	36.72 [36.44, 37.00]	36.61 [36.00, 37.17]	<0.001	0.142
Respiratory rate,/min		18.00 [16.00, 22.00]	19.00 [16.00, 22.00]	17.00 [14.00, 21.00]	<0.001	0.175
SpO2, %		98.00 [96.00, 100.00]	98.00 [96.00, 100.00]	99.00 [97.00, 100.00]	<0.001	0.219
Bicarbonate, mEq/L		22.00 [20.00, 24.00]	24.60 [22.40, 26.00]	24.00 [22.00, 26.00]	<0.001	0.396
Creatinine, mg/dL		0.90 [0.70, 1.30]	0.91 [0.74, 1.20]	0.90 [0.70, 1.20]	0.008	0.070
Blood urea nitrogen, mg/dL		17.00 [13.00, 26.00]	17.00 [12.00, 24.00]	18.00 [13.00, 26.00]	<0.001	0.095
Sodium, mEq/L		139.00 [136.00, 141.00]	139.00 [137.00, 141.00]	139.00 [136.00, 141.00]	<0.001	0.098
Potassium, mEq/L		4.10 [3.80, 4.50]	3.90 [3.64, 4.20]	3.90 [3.60, 4.30]	<0.001	0.246
Chloride, mEq/L		104.00 [101.00, 107.00]	105.00 [103.00, 108.00]	106.00 [103.00, 109.00]	<0.001	0.278
Blood glucose, mg/dL		129.00 [104.00, 166.00]	124.00 [102.00, 156.00]	134.00 [109.00, 169.00]	<0.001	0.136
White blood cell, 10^9^/L		10.20 [7.80, 13.70]	9.60 [7.60, 12.30]	10.94 [8.40, 13.80]	<0.001	0.154
Hemoglobin, g/dL		11.40 [9.60, 13.00]	12.30 [11.00, 13.60]	11.25 [10.00, 12.70]	<0.001	0.280
Hematocrit, %		35.00 [29.70, 39.50]	36.90 [33.14, 40.59]	33.10 [29.17, 37.10]	<0.001	0.389
Platelet, 10^9^/L		197.90 [150.00, 251.00]	208.00 [171.00, 251.00]	209.00 [157.75, 269.00]	<0.001	0.104
Calcium, mg/dL		8.60 [8.20, 9.10]	8.60 [8.28, 9.00]	8.46 [8.00, 8.90]	<0.001	0.174
Mechanical ventilation	No	2177 (70.2)	2319 (73.0)	414 (53.4)	<0.001	0.277
	Yes	923 (29.8)	856 (27.0)	362 (46.6)		
Renal replacement therapy	No	3038 (98.0)	3130 (98.6)	760 (97.9)	0.164	0.033
	Yes	62 (2.0)	45 (1.4)	16 (2.1)		
Vasopressor use	No	2408 (77.7)	2860 (90.1)	515 (66.4)	<0.001	0.399
	Yes	692 (22.3)	315 (9.9)	261 (33.6)		
Sedative use	No	2187 (70.5)	2553 (80.4)	489 (63.0)	<0.001	0.262
	Yes	913 (29.5)	622 (19.6)	287 (37.0)		
Modified rankin scale at discharge	0	54 (1.7)	92 (2.9)	7 (0.9)	<0.001	0.394
	1	133 (4.3)	209 (6.6)	25 (3.2)		
	2	332 (10.7)	462 (14.6)	37 (4.8)		
	3	717 (23.1)	781 (24.6)	115 (14.8)		
	4	779 (25.1)	733 (23.1)	222 (28.6)		
	5	610 (19.7)	529 (16.7)	182 (23.5)		
	6 (Death)	475 (15.3)	369 (11.6)	188 (24.2)		
Hospital mortality	Survived	2625 (84.7)	2806 (88.4)	588 (75.8)	<0.001	0.222
	Died	475 (15.3)	369 (11.6)	188 (24.2)		

Abbreviations: BP, Blood pressure; NIHSS, National Institutes of Health Stroke Scale; LVO, Large vessel occlusion; EVT, Endovascular thrombectomy; TOAST, Trial of ORG 10172 in Acute Stroke Treatment; MRS, Modified Rankin Scale; SAPS II, Simplified Acute Physiology Score II; OASIS, Oxford Acute Severity of Illness Score; SOFA, Sequential Organ Failure Assessment; GCS, Glasgow Coma Scale; SMD, Standardized mean difference.

Continuous variables are presented as median [IQR]. Categorical variables are presented as n (%).

Note: MIMIC-IV was used for model development; eICU and ChineseICU were used for external validation.

TOAST classification: Large artery atherosclerosis (LAA), Cardioembolism (CE), Small vessel occlusion (SVO), Other determined etiology, and Undetermined etiology.

Modified Rankin Scale: 0 = no symptoms; 1 = no significant disability; 2 = slight disability; 3 = moderate disability; 4 = moderately severe disability; 5 = severe disability; 6 = death.

The six trajectory variables (SBP, DBP, MAP, Heart Rate, Respiratory Rate, SpO2) used for subtype classification are presented separately in the trajectory analysis tables.

**Fig. 2:**
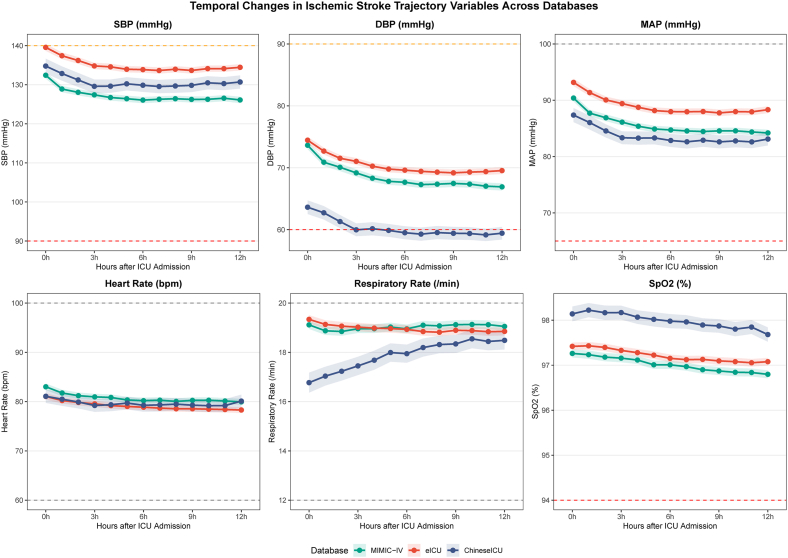
**Temporal Trends of Trajectory Variables Across Databases.** Line plots showing the temporal changes in six vital sign trajectory variables (systolic blood pressure, diastolic blood pressure, mean arterial pressure, heart rate, respiratory rate, and peripheral oxygen saturation) from 0 to 12 h after ICU admission across three databases. Solid lines represent mean values, and shaded areas indicate 95% confidence intervals. Reference lines indicate clinically relevant thresholds.

### Identification of vital sign trajectory phenotypes

Based on the MIMIC-IV development cohort, GBMTM analysis was performed to identify distinct vital sign trajectory phenotypes among ischemic stroke patients. Model comparison across K = 2 to K = 7 clusters revealed that the three-cluster solution provided the optimal balance between model fit and clinical interpretability, supported by the lowest AIC (12746.92) and BIC (13838.80), the highest average silhouette coefficient (0.174), the maximum Calinski-Harabasz index (742.93), and the largest gap statistic (1.902) (Supplementary Fig. 1 and Fig. 3A). The three identified phenotypes demonstrated distinct hemodynamic and respiratory patterns during the first 12 h after ICU admission (Fig. 3B). Subtype 1 (n = 795, 25.6%), designated as the tachycardic-tachypneic phenotype, was characterized by moderate baseline blood pressure with a marked declining trend (SBP: 121  →  113 mmHg; DBP: 71  →  63 mmHg), the highest heart rate (99  →  93 bpm), and elevated respiratory rate (22/min), suggesting heightened sympathetic activation with progressive hemodynamic deterioration. Subtype 2 (n = 1,163, 37.5%), designated as the hypertensive-stable phenotype, exhibited the highest and most stable blood pressure levels throughout the observation period (SBP: 149  →  143 mmHg; DBP: 86  →  79 mmHg; MAP: 103  →  98 mmHg), with moderate heart rate (82  →  79 bpm) and the stable SpO2 levels (96.5–96.9%), indicating preserved cardiovascular reserve. Subtype 3 (n = 1,142, 36.8%), designated as the low-diastolic quiescent phenotype, displayed the lowest diastolic blood pressure values (DBP: 64  →  57 mmHg; MAP: 81  →  75 mmHg), lowest heart rate (73  →  71 bpm), and lowest respiratory rate (17–18/min), with the highest SpO2 levels (97.1–98.0%), potentially reflecting reduced metabolic demand or autonomic dysfunction.

**Fig. 3:**
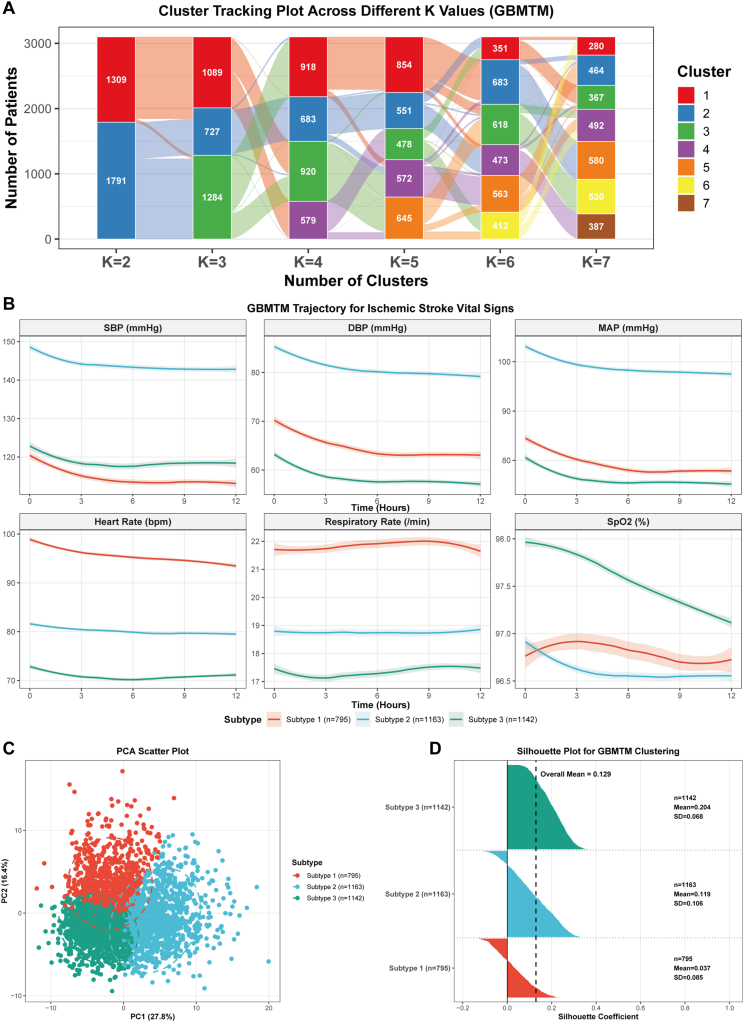
**Identification and Characterization of Ischemic Stroke Phenotypes.** (A) Alluvial plot showing cluster membership tracking across different numbers of clusters (K = 2 to K = 7). (B) Trajectory plots displaying the temporal patterns of six vital signs for each identified phenotype, with solid lines representing mean values and shaded areas representing 95% confidence intervals. (C) Principal component analysis scatter plot with 95% confidence ellipses showing the separation of three phenotypes. (D) Silhouette plot demonstrating clustering quality for each phenotype with mean silhouette coefficients.

Principal component analysis demonstrated visible separation among the three phenotypes in two-dimensional space (Fig. 3C). Clustering quality assessment revealed an overall mean silhouette coefficient of 0.128, with Subtype 3 showing the highest clustering coherence (mean silhouette: 0.204 ± 0.068) and no misclassified patients, followed by Subtype 2 (0.119 ± 0.106, 16.3% negative silhouette values) and Subtype 1 (0.037 ± 0.085, 36.6% negative silhouette values) (Fig. 3D). The relatively low overall silhouette coefficient is consistent with the continuous nature of vital sign data, where phenotypes represent gradual transitions rather than discrete boundaries; nevertheless, bootstrap stability analysis with 1000 iterations demonstrated robust phenotype assignments, with 92% of Subtype 1 patients, 89% of Subtype 2 patients, and 94% of Subtype 3 patients being consistently assigned to the same phenotype across resampling iterations.

### Prognostic value of phenotypes and comparison with traditional severity scores

The three identified phenotypes demonstrated significantly different mortality outcomes across all time points. Kaplan-Meier survival analysis revealed that Subtype 1 (tachycardic-tachypneic phenotype) had the highest mortality rates, followed by Subtype 3 (low-diastolic quiescent phenotype) and Subtype 2 (hypertensive-stable phenotype) with the lowest mortality (all log-rank P < 0.001; Fig. 4A–C). In-hospital mortality rates were 29.7% (236/795) for Subtype 1, 14.9% (170/1142) for Subtype 3, and 5.9% (69/1163) for Subtype 2. Similar patterns were observed for 28-day mortality (38.6%, 19.3%, and 7.7%, respectively) and one-year mortality (64.4%, 32.2%, and 12.9%, respectively). Multivariable Cox regression analysis confirmed that phenotype classification remained an independent predictor of mortality after adjustment for demographics, severity scores, laboratory values, and interventions (Supplementary Table 2). In the fully adjusted model (Model 4), compared with Subtype 2, Subtype 1 showed significantly elevated risks for in-hospital mortality (HR 3.55, 95% CI 2.23–5.64, P < 0.001), 28-day mortality (HR 4.38, 95% CI 2.97–6.46, P < 0.001), and one-year mortality (HR 4.50, 95% CI 3.35–6.04, P < 0.001). Subtype 3 also demonstrated increased risks compared with Subtype 2 (in-hospital: HR 1.77, 95% CI 1.12–2.80, P = 0.014; 28-day: HR 2.29, 95% CI 1.55–3.37, P < 0.001; one-year: HR 1.96, 95% CI 1.46–2.64, P < 0.001).

**Fig. 4:**
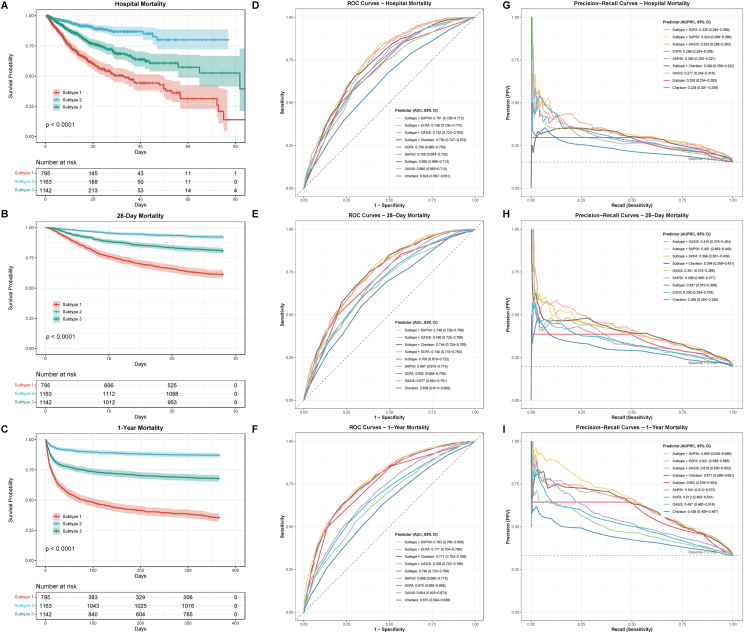
**Prognostic Value of Phenotypes and Comparison with Traditional Severity Scores.** Kaplan-Meier survival curves for (A) in-hospital mortality, (B) 28-day mortality, and (C) one-year mortality stratified by phenotype, with log-rank P values and number at risk tables. Receiver operating characteristic (ROC) curves comparing the discriminative ability of phenotype-based prediction versus traditional severity scores (SOFA, SAPS II, OASIS, Charlson comorbidity index) and their combined models for (D) in-hospital mortality, (E) 28-day mortality, and (F) one-year mortality, with area under the ROC curve values and 95% confidence intervals. Precision-recall curves addressing class imbalance for (G) in-hospital mortality, (H) 28-day mortality, and (I) one-year mortality, with area under the precision-recall curve values and 95% confidence intervals. Dashed horizontal lines indicate baseline prevalence.

**Fig. 5:**
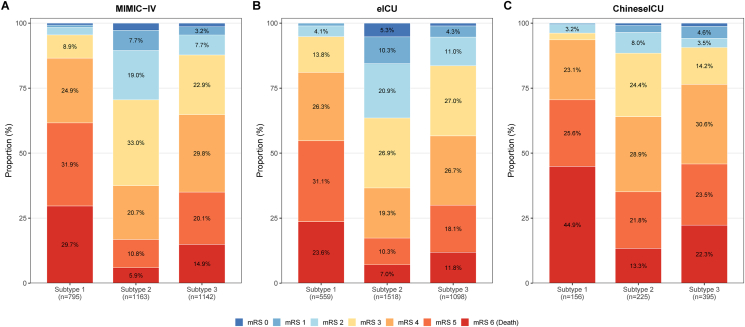
**Distribution of Modified Rankin Scale Scores at Discharge by Phenotype Across Three Databases.** Stacked bar charts showing the distribution of modified Rankin Scale (MRS) scores (0–6) at discharge for each phenotype in (A) MIMIC-IV, (B) eICU, and (C) Chinese critical care database. Colors range from blue (MRS 0, no symptoms) to red (MRS 6, death). Kruskal-Wallis tests with Dunn's pairwise comparisons (Bonferroni-corrected) are displayed for each database. Sample sizes are shown beneath each phenotype label.

Using phenotype as the sole predictor, discriminative performance analysis showed that phenotype-based prediction achieved AUROCs of 0.690 for in-hospital mortality, 0.700 for 28-day mortality, and 0.740 for one-year mortality (Fig. 4D–F). Corresponding AUPRCs were 0.259 (95% CI 0.234–0.283), 0.337 (95% CI 0.310–0.366), and 0.562 (95% CI 0.535–0.594), respectively (Fig. 4G–I). DeLong test comparisons revealed that phenotype classification significantly outperformed the Charlson comorbidity index across all outcomes (all P < 0.001; Supplementary Table 3). For one-year mortality, phenotype-based prediction demonstrated superior discrimination compared with all traditional severity scores, including SOFA (ΔAUC = 0.065, P < 0.0001), SAPS II (ΔAUC = 0.042, P = 0.0006), and OASIS (ΔAUC = 0.087, P < 0.0001). Combining phenotype with traditional scores further improved AUPRC, with the highest values observed for Subtype + SAPS II in one-year mortality prediction (AUPRC = 0.655, 95% CI 0.628–0.686). Incremental value analysis demonstrated that adding phenotype classification to traditional scores significantly improved prediction performance (Supplementary Table 4). For one-year mortality, combining phenotype with Charlson index yielded the highest improvement (NRI = 0.140, 95% CI 0.114–0.170, P < 0.0001; IDI = 0.052, 95% CI 0.043–0.060, P < 0.0001). Calibration assessment showed that phenotype-based predictions were well-aligned with observed probabilities, and decision curve analysis confirmed positive net benefit across a range of clinically relevant threshold probabilities for all mortality outcomes (Supplementary Fig. 2). Beyond mortality outcomes, functional status at discharge also differed significantly across phenotypes. The distribution of modified Rankin Scale scores showed progressively worse functional outcomes from Subtype 2 to Subtype 3 to Subtype 1 across all three databases (all Kruskal-Wallis P < 0.001; Fig. 5).

Baseline clinical characteristics stratified by phenotype further revealed distinct clinical profiles (Supplementary Table 5 and Supplementary Fig. 3). Compared with the hypertensive-stable phenotype, the tachycardic-tachypneic phenotype exhibited higher NIHSS scores (18.0 vs. 12.0), greater prevalence of large vessel occlusion (55.1% vs. 29.3%), higher SOFA scores (5.0 vs. 2.0), and more frequent use of mechanical ventilation (39.9% vs. 15.5%) and vasopressors (31.4% vs. 8.2%), while the low-diastolic quiescent phenotype showed intermediate severity with the oldest age (74.0 years) and the lowest hemoglobin levels (10.9 g/dL) (all P < 0.001).

### Subgroup analysis of phenotype-mortality association

To evaluate the robustness of the phenotype-mortality association, subgroup analyses were performed across 14 clinical subgroups stratified by demographics, stroke characteristics, disease severity, and interventions (Supplementary Fig. 4). Subtype 1 showed consistently higher in-hospital mortality compared with Subtype 2 across nearly all subgroups (HR range: 2.56–9.35), with particularly strong prognostic value in the endovascular thrombectomy subgroup (Subtype 1 vs. Subtype 2 [reference]: HR 8.13, 95% CI 2.86–23.14, P < 0.001). Significant interaction effects were observed for age (P = 0.012), SOFA score (P = 0.001), GCS (P < 0.001), and Charlson comorbidity index (P = 0.001), while no significant interactions were found for stroke-specific characteristics including NIHSS (P = 0.943), LVO status (P = 0.296), EVT (P = 0.223), or TOAST classification (P = 0.814), supporting the generalizability of phenotype prognostication across different stroke subtypes.

### Treatment response heterogeneity across phenotypes

**Fig. 6:**
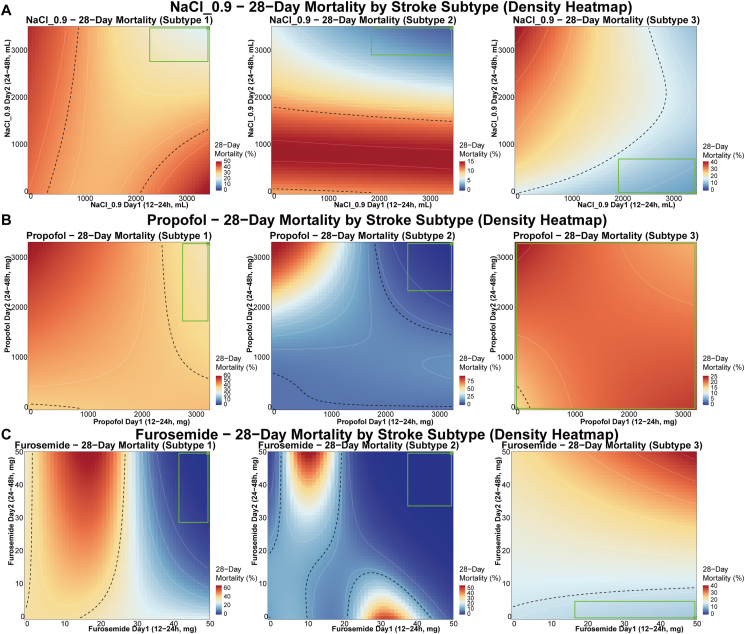
**Treatment-Response Heterogeneity: Dose-Response Heatmaps.** Two-dimensional density heatmaps displaying predicted 28-day mortality across different dose combinations for each phenotype: (A) normal saline, (B) propofol, and (C) furosemide. Green rectangles indicate optimal dose regions corresponding to the lowest 5% of predicted mortality, and asterisks mark the optimal dose combination.

Given the distinct prognostic profiles of the three phenotypes, we further explored whether treatment-mortality associations differed across phenotypes using GAM analysis. Three commonly used ICU treatments were examined: normal saline, propofol, and furosemide. The predicted 28-day mortality surfaces revealed substantial heterogeneity in dose-mortality associations across phenotypes (Fig. 6, Supplementary Fig. 5). For normal saline administration, Subtype 1 (tachycardic-tachypneic phenotype) and Subtype 2 (hypertensive-stable phenotype) showed the lowest predicted mortality with higher fluid volumes on both days (Day 1: 2357–3500 mL and 1929–3500 mL; Day 2: 2786–3500 mL and 2929–3500 mL, respectively), achieving minimum predicted mortality rates of 19.64% and 1.17% (Fig. 6A). In contrast, Subtype 3 (low-diastolic quiescent phenotype) demonstrated a divergent pattern, with the lowest predicted mortality at Day 1 dosing of 2000–3500 mL but restricted Day 2 vol of 0–714 mL.

Propofol administration showed similar phenotype-specific patterns (Fig. 6B). Subtypes 1 and 2 showed the lowest predicted mortality at higher doses (Day 1 ranges: 2829–3300 mg and 2492–3300 mg, respectively), while Subtype 3 showed the lowest predicted mortality (15.68%) with minimal or no propofol exposure (0 mg on both days). For furosemide, all three phenotypes showed the lowest predicted mortality at higher Day 1 doses (42–50 mg for Subtype 1, 38–50 mg for Subtype 2, and 17–50 mg for Subtype 3), but Subtype 3 again required restricted Day 2 dosing (0–5 mg) compared with continued administration in other phenotypes (Fig. 6C). Notably, actual medication utilization rates were relatively low across all phenotypes, with propofol use ranging from 8.8% to 30.9% and furosemide use ranging from 3.5% to 11.5% on Day 1.

### External validation of phenotypes

**Fig. 7:**
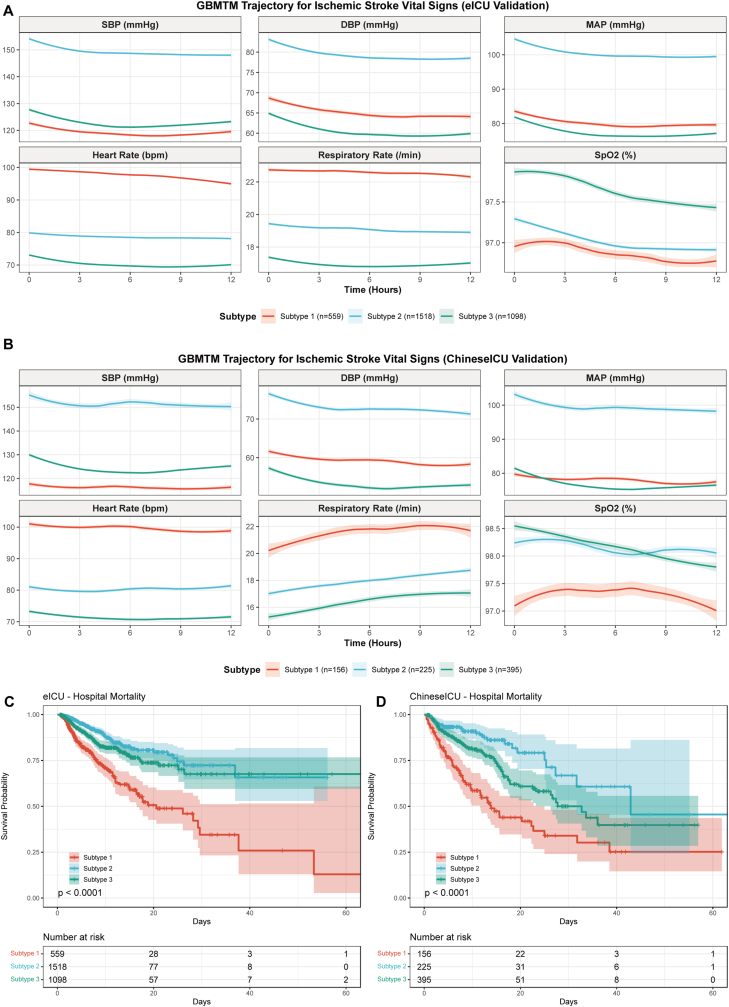
**External Validation of Phenotypes.** Trajectory plots showing vital sign patterns for (A) eICU and (B) Chinese critical care database validation cohorts. Kaplan-Meier survival curves for in-hospital mortality in (C) eICU and (D) Chinese critical care database, demonstrating consistent prognostic discrimination of phenotypes across external populations.

To assess the generalizability of the identified phenotypes, external validation was performed in the eICU (n = 3175) and Chinese critical care database (n = 776) cohorts. The phenotype assignment based on trajectory mean squared error demonstrated consistent distribution patterns across databases, with Subtype 2 (hypertensive-stable) being the most prevalent in eICU (47.8%) and Subtype 3 (low-diastolic quiescent) being most common in the Chinese cohort (50.9%) (Table 1). The vital sign trajectory patterns in both validation cohorts closely resembled those observed in the MIMIC-IV development cohort, with preserved inter-phenotype differences in blood pressure, heart rate, and respiratory rate profiles (Fig. 7A–B).

The prognostic discrimination of phenotypes was successfully validated in both external cohorts. In the eICU cohort, in-hospital mortality rates were 23.6% for Subtype 1, 11.8% for Subtype 3, and 7.0% for Subtype 2 (Fig. 7C). Cox regression analysis confirmed that Subtype 1 (HR 3.14, 95% CI 2.43–4.06, P < 0.001) and Subtype 3 (HR 1.55, 95% CI 1.20–2.00, P < 0.001) had significantly higher mortality compared with Subtype 2. Similar results were observed in the Chinese cohort, where mortality rates were 44.9% for Subtype 1, 22.3% for Subtype 3, and 13.3% for Subtype 2 (Fig. 7D). The hazard ratios remained significant for Subtype 1 (HR 3.80, 95% CI 2.46–5.86, P < 0.001) and Subtype 3 (HR 1.84, 95% CI 1.21–2.79, P = 0.005) compared with Subtype 2, confirming the robust prognostic value of the phenotype classification across diverse patient populations and healthcare settings.

## Discussion

In this multicenter retrospective cohort study utilizing three large critical care databases, we identified three distinct vital sign trajectory phenotypes among critically ill patients with ischemic stroke during the first 12 h after ICU admission. The tachycardic-tachypneic phenotype (Subtype 1) was characterized by moderate baseline blood pressure with progressive decline, the highest heart rate, and elevated respiratory rate; the hypertensive-stable phenotype (Subtype 2) exhibited the highest and most stable blood pressure levels with preserved cardiovascular reserve; and the low-diastolic quiescent phenotype (Subtype 3) displayed the lowest diastolic blood pressure values, lowest heart rate, and lowest respiratory rate, potentially reflecting reduced metabolic demand or autonomic dysfunction. These phenotypes demonstrated significantly different mortality outcomes, with Subtype 1 showing the highest in-hospital mortality (29.7%), followed by Subtype 3 (14.9%) and Subtype 2 (5.9%). Importantly, the phenotype classification remained an independent predictor of mortality after adjustment for demographics, severity scores, laboratory values, and interventions, and was successfully validated in two external cohorts from the eICU and Chinese critical care databases. Furthermore, we observed substantial heterogeneity in dose-mortality associations across phenotypes for normal saline, propofol, and furosemide, which may inform future investigation of precision-guided therapeutic approaches in critically ill stroke patients.

Our finding that blood pressure trajectories are associated with mortality in ischemic stroke patients is consistent with previous studies. Katsanos et al. demonstrated that high-SBP trajectories after endovascular thrombectomy were associated with higher odds of neurological deterioration, intracranial hemorrhage, and mortality [4], and Lee et al. found that slowly dropping SBP trajectories were linked to worse composite cardiovascular outcomes [5]. However, our study extends these findings by incorporating multiple vital signs simultaneously using group-based multi-trajectory modeling, which better captures the complex interrelationships among hemodynamic parameters in ICU settings. Multiple randomized trials have consistently demonstrated that intensive blood pressure lowering after thrombectomy is associated with worse functional outcomes. The BEST-II trial suggested a low probability of benefit from aggressive BP lowering [14], and subsequent trials reinforced this finding: the ENCHANTED2/MT trial was stopped early after intensive SBP control to lower than 120 mmHg led to worse functional outcomes [15], and the OPTIMAL-BP trial was similarly terminated due to safety concerns with intensive BP management [16]. Secondary analyses of these trials identified several effect modifiers including intracranial atherosclerotic stenosis [17], white matter hyperintensity burden [18], collateral status [19], prior intravenous thrombolysis [20], and multiple thrombectomy attempts [21], while threshold-based analysis demonstrated that both prolonged hypoperfusion and extreme surges were associated with worse outcomes [22]. Meta-analyses confirmed that intensive BP control after thrombectomy reduced functional independence without reducing hemorrhage or mortality [[23], [24], [25]]. Recent advances further support individualized approaches: an autoregulation-guided study demonstrated that deviations from personalized BP thresholds were associated with secondary brain injury [26], and a Lancet Neurology review concluded that intensive BP lowering to SBP <140 mmHg should be avoided before and after reperfusion therapy [27]. Collectively, these findings support a paradigm shift toward individualized, phenotype-specific hemodynamic management rather than uniform blood pressure targets.

The prognostic significance of heart rate dynamics observed in our study aligns with existing evidence from acute ischemic stroke populations. Feng et al. identified four heart rate trajectories during the first 7 days after stroke onset and found that sustained high heart rate trajectory was significantly associated with a higher risk of poor functional outcome at 3 months (OR 3.00) compared to markedly decreasing heart rate trajectory [6]. Lee et al. further demonstrated that maximum heart rate during the acute period was the best predictor of major clinical events, with an estimated cutoff point of 100 beats per minute [28]. In patients with atrial fibrillation, a nonlinear J-shaped association was observed between mean heart rate and mortality [29]. Additionally, heart rate turbulence, an ECG-based marker of autonomic cardiac regulation, has been associated with increased disability and newly detected atrial fibrillation after acute ischemic stroke [30]. Our tachycardic-tachypneic phenotype, characterized by the highest heart rate values, demonstrated the worst outcomes. The tachycardia observed in this phenotype may reflect autonomic dysfunction, but could also represent an appropriate physiological response to hypovolemia, systemic inflammation, or other stressors, and its precise mechanism warrants further investigation. Kamieniarz-Mędrygał et al. reported that pulse pressure variability during the first 24 h after admission was robustly associated with 30-day unfavorable outcomes, with thresholds of 14 mmHg for standard deviation and 26 mmHg for maximal successive change [31], suggesting that both the level and variability of hemodynamic parameters contribute to prognosis.

The application of group-based trajectory modeling to characterize phenotypes in critically ill stroke patients represents a methodological advancement that has been increasingly utilized in recent studies. Tang et al. employed this approach to identify three fluid balance trajectory patterns in elderly patients with acute ischemic stroke and found that high fluid balance was an independent risk factor for in-hospital mortality with a 1.57-fold increased risk of hospital mortality associated with fluid overload [7]. Li et al. identified four blood glucose level trajectories within 24 h of admission and demonstrated that moderate level-stable trajectory was associated with increased 30-day mortality risk compared with low level-stable trajectory (HR 1.28) [8]. Zou et al. characterized serum sodium trajectories within 72 h of admission and found that rapid fluctuations were associated with elevated mortality risk at multiple timepoints (HRs ranging from 1.99 to 2.51) [10]. Zhao et al. further demonstrated that a consistent decreasing trajectory of serum lactate concentration was associated with an increased risk of mortality in ischemic stroke patients [9]. Our study builds upon these single-parameter trajectory analyses by simultaneously modeling six vital signs, thereby providing a more comprehensive characterization of patient phenotypes. This approach is analogous to recent work by Wang et al., who developed vital sign trajectory-based subphenotypes in acute pancreatitis patients and identified four distinct subtypes with differential mortality risks and treatment responses [32].

The treatment response heterogeneity observed across phenotypes in our study has important implications for precision critical care. We found that higher fluid volumes were associated with lower predicted mortality in Subtype 1 and Subtype 2 on both days, whereas Subtype 3 showed a divergent pattern suggesting that continued aggressive fluid administration may not be associated with improved outcomes in this phenotype. These findings are consistent with emerging evidence supporting phenotype-guided fluid therapy in critically ill patients. Kiernan et al. recently demonstrated that molecular subphenotypes in sepsis respond differently to fluid resuscitation strategy, with the hyperinflammatory phenotype showing higher mortality with liberal versus restrictive fluid strategy (41% vs. 27%) while the hypoinflammatory phenotype showed no difference [33]. The differential responses to propofol observed in our study are also clinically relevant, as propofol has been shown to possess neuroprotective properties in cerebral ischemia/reperfusion injury through immunomodulatory mechanisms [34]. Sousa et al. compared dexmedetomidine and propofol in experimental acute ischemic stroke and found that dexmedetomidine, but not propofol, induced brain and lung protection [35]. Notably, the post-thrombectomy subgroup in our study demonstrated particularly strong phenotype-mortality associations (Subtype 1 vs. Subtype 2 [reference]: HR 8.13, 95% CI 2.86–23.14), suggesting that vital sign trajectory phenotyping may be especially relevant in this population where blood pressure management remains controversial [36]. The INDIVIDUATE study found no significant difference between individualized and standardized blood pressure management during endovascular treatment [37], suggesting that patient phenotype may be a more important determinant of optimal management than absolute blood pressure targets.

This study has several strengths. First, we utilized three large, geographically diverse critical care databases, providing robust external validation across different healthcare systems and patient populations. The consistent prognostic discrimination of phenotypes across MIMIC-IV, eICU, and the Chinese critical care database supports the generalizability of our findings. Second, the group-based multi-trajectory modeling approach enabled simultaneous characterization of multiple vital sign patterns, capturing the complex hemodynamic interactions that single-parameter analyses cannot address. Third, we performed comprehensive analyses including bootstrap stability assessment, clustering quality evaluation, and progressive multivariable adjustment to ensure the robustness of our phenotype classification. Fourth, the treatment response heterogeneity analysis using generalized additive models provides novel insights into phenotype-specific optimal dosing strategies. However, several limitations should be acknowledged. The retrospective nature of the study precludes causal inference, and unmeasured confounders may influence the observed associations. In particular, the treatment-mortality associations observed across phenotypes should be interpreted as exploratory findings, as variations in treatment doses likely reflect underlying disease severity and clinical decision-making rather than direct therapeutic effects. The 12-h observation window may not capture the full spectrum of hemodynamic changes during ICU admission. Medication utilization rates were relatively low across all phenotypes, limiting the power of treatment response analyses. Additionally, the phenotype assignment for external validation cohorts was based on trajectory similarity rather than de novo clustering, which may introduce classification bias.

Our findings have important clinical implications for risk stratification and treatment individualization in critically ill ischemic stroke patients. The identification of distinct vital sign trajectory phenotypes within the first 12 h after ICU admission provides an early prognostic framework that can guide clinical decision-making. The observation that phenotype classification outperformed traditional severity scores such as the Charlson comorbidity index for one-year mortality prediction suggests potential utility in long-term prognostication. The differential treatment responses across phenotypes support the emerging paradigm of precision critical care medicine, wherein treatment strategies are tailored based on patient subtype rather than one-size-fits-all approaches. Future prospective studies are warranted to validate these phenotypes in real-time clinical settings and to evaluate whether phenotype-guided treatment protocols can improve patient outcomes. Additionally, investigation of the underlying pathophysiological mechanisms distinguishing these phenotypes, including autonomic function, inflammatory responses, and cardiac-brain interactions, may provide targets for therapeutic intervention. Integration of these vital sign trajectory phenotypes with biomarker profiles and imaging findings may further refine patient stratification and enable truly personalized critical care for ischemic stroke patients.

In this multicenter retrospective cohort study, we identified three distinct vital sign trajectory phenotypes among critically ill patients with ischemic stroke during the first 12 h after ICU admission: tachycardic-tachypneic, hypertensive-stable, and low-diastolic quiescent phenotypes. These phenotypes demonstrated significantly different mortality outcomes, with the tachycardic-tachypneic phenotype exhibiting the highest mortality rates and the hypertensive-stable phenotype showing the most favorable prognosis. The phenotype classification provided independent prognostic value after multivariable adjustment and was successfully validated in two external cohorts. Furthermore, substantial treatment response heterogeneity was observed across phenotypes for normal saline, propofol, and furosemide administration. These findings support the potential for vital sign trajectory-based phenotyping to guide early risk stratification and precision therapeutic approaches in critically ill ischemic stroke patients.

## Ethics statement

This study was conducted in accordance with the principles of the Declaration of Helsinki and adhered to the ethical guidelines for secondary analysis of de-identified public datasets. This study utilized three publicly available critical care databases: MIMIC-IV, eICU Collaborative Research Database, and the Chinese Critical Care Database, all accessed through PhysioNet. These databases contain de-identified health information that was collected under appropriate Institutional Review Board (IRB) approval. As this study involved only retrospective analysis of de-identified data from publicly available sources, no additional ethical approval was required. All investigators completed the CITI Data or Specimens Only Research training required by PhysioNet and signed data use agreements prior to accessing the databases (Record ID: 67424917).

## Consent for publication

Not applicable.

## Availability of data and materials

The datasets analyzed in this study are publicly available through PhysioNet (https://physionet.org/): MIMIC-IV (https://physionet.org/content/mimiciv/3.1/), eICU Collaborative Research Database (https://physionet.org/content/eicu-crd/2.0/), and Chinese Critical Care Database (https://physionet.org/content/zhejiang-ehr-critical-care/1.0/).

## Author contributions

L.W. and D.W. contributed equally to this work as co-first authors. Conceptualization: J.H. and J.D.; Data curation: B.T. and D.W.; Formal analysis: L.W. and D.W.; Investigation: L.W., D.W., and B.T.; Methodology: L.W., D.W., and J.H.; Software: D.W. and B.T.; Supervision: J.H. and J.D.; Validation: B.T. and J.D.; Visualization: L.W. and D.W.; Writing – original draft: L.W. and D.W.; Writing – review and editing: J.H. and J.D.; Project administration: J.H. and J.D. All authors read and approved the final manuscript.

## Human ethics and consent to participate

Not applicable.

## Funding

Not applicable.

## Declaration of competing interest

The authors declare that they have no competing interests.

## Figures and Tables

**figs1:**
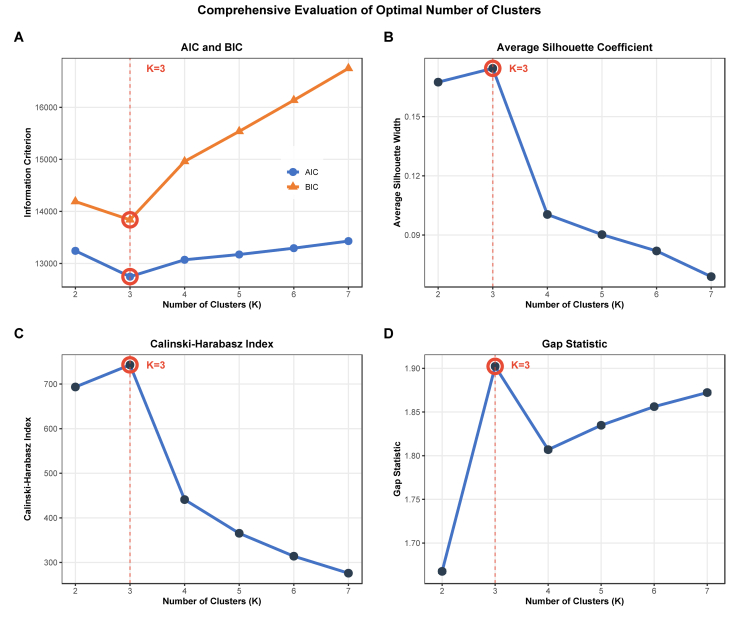


**figs2:**
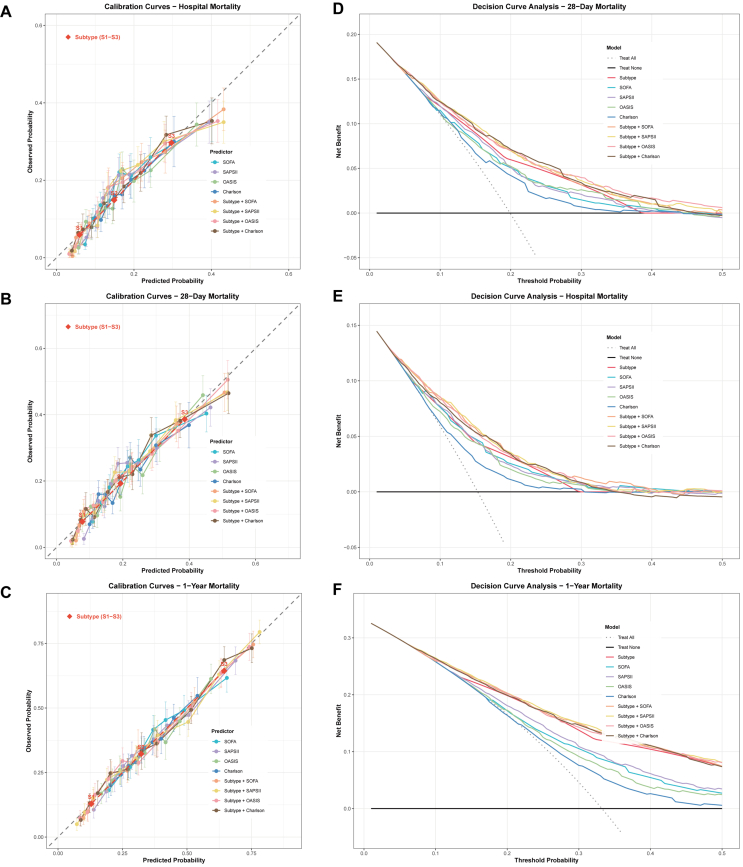


**figs3:**
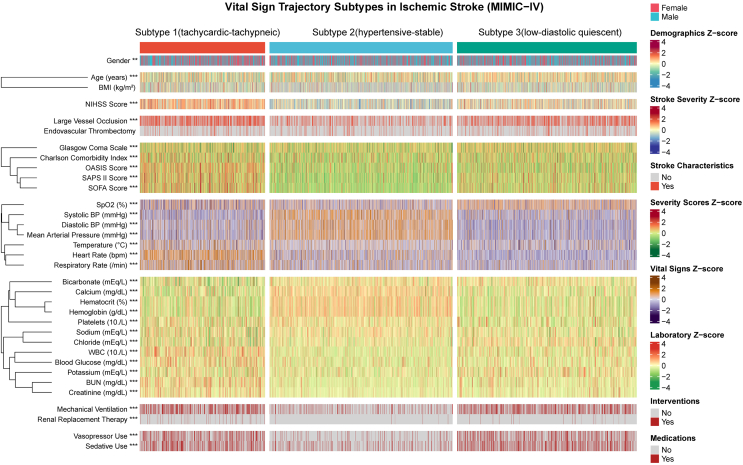


**figs4:**
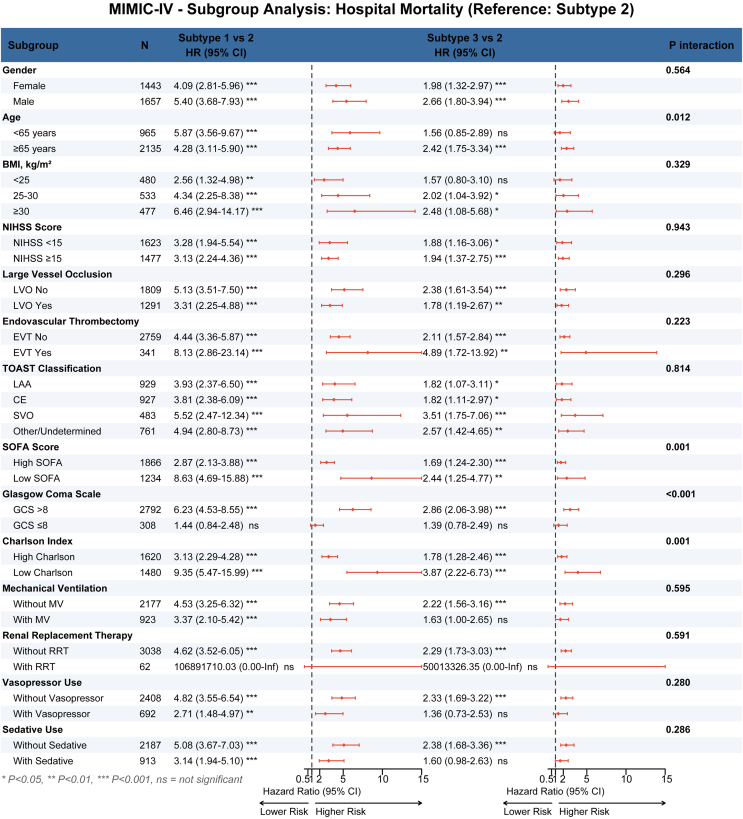


**figs5:**